# P64 Seroprevalence of anti-measles, anti-rubella, anti-varicella and anti-HBs antibodies at enrolment among healthcare workers at the Emirates Hospital Jumeirah, Dubai, UAE

**DOI:** 10.1093/jacamr/dlaf230.071

**Published:** 2025-12-04

**Authors:** Francesca Cainelli, Mohamad Chamas, Avany Gireesh Kumar, Wajiha Amin, Hady Elkhodary, Hussain Nasser Saleh Alrahma, Wael Sammur

**Affiliations:** Emirates Hospital Jumeirah, Dubai, UAE; Emirates Specialty Hospital, Dubai, UAE; Emirates Hospital Jumeirah, Dubai, UAE; Emirates Specialty Hospital, Dubai, UAE; Emirates Hospital Jumeirah, Dubai, UAE; Emirates Hospital Jumeirah, Dubai, UAE; Emirates Hospital Jumeirah, Dubai, UAE; Emirates Hospital Jumeirah, Dubai, UAE; Emirates Hospital Jumeirah, Dubai, UAE

## Abstract

**Background:**

People working in medical facilities have a greater risk of being exposed to and acquiring hepatitis B, measles, rubella and varicella. A seroprevalence survey for antibodies to these viruses, followed by an adequate vaccination programme is necessary to diminish the risk of vaccine- preventable diseases for other workers and patients in hospital settings, and is recommended by Dubai Health Authority. Vaccines for hepatitis B, measles, rubella and varicella are provided free of charge to susceptible licensed healthcare workers (HCWs) at Emirates Hospital Jumeirah in Dubai, UAE. Administrative staff are screened and vaccinated against hepatitis B only. HCWs exhibiting a negative or equivocal antibody titre by EIA are considered as susceptible and recommended for vaccination.

**Objectives:**

To conduct a seroprevalence survey on antibodies to hepatitis B surface antigen, measles, rubella and varicella in licensed healthcare workers (HCWs) and administrative staff at Emirates Hospital Jumeirah, Dubai, UAE at the time when they are enrolled.

**Patients and methods:**

A total of 347 licensed HCWs (238, 68.5% females; age 22–79 years, mean 39) and 119 administrative staff (70%–58.8% females; age 22–63 years, mean 37) were surveyed between 2021 and 2025. Commercially available EIA kits were used to detect specific IgG antibodies against hepatitis B surface antigen, measles, rubella and varicella. The cut-off values for seronegativity were <10 IU/mL for HBsAb, either <1.2 IU/mL or <16.5 IU/mL or <275 IU/mL for measles, either <10 IU/mL or <12 IU/mL for rubella and either <1.2 IU/mL or <110 IU/mL or <150 IU/mL for varicella, depending on the assays used which changed over time.

**Results:**

Of the licensed HCWs tested, 91.6 % were HBsAb-positive, 89 % positive for anti-rubella, 88% for anti-measles and 85 % for anti-varicella antibodies. Of the administrative staff, 88% were HBsAb- positive, and females were more frequently seropositive than males (90% versus 86%), but the difference was not statistically significant. Female HCWs were significantly more frequently HBsAb-positive than males (95.8% versus 83.3%, *P<*0.001); in contrast, male HCWs were more often seropositive to varicella (93% versus 81%, *P<*0.01). HCWs aged 30–39 years were more frequently seropositive (47.4%) than those below 29 years of age (10.7%) or those aged 40–49 years (27.5%).

**Conclusions:**

Not all HCWs at enrolment at a private hospital in Dubai are immunized against hepatitis B, rubella, measles or varicella. Vaccinations are therefore very important to reach a 100% immunization target. While a higher frequency of HBsAb seropositivity in females was expected, the unexpected higher prevalence of immunity to varicella in male HCWs may be explained by concurrent factors (e.g. reduced prevalence of obesity) that were not included in our database.

